# Improvements in Prescribing Indicators and Antibiotic Utilization Patterns Following Antimicrobial Stewardship Intervention at a District Hospital in Ghana

**DOI:** 10.3390/tropicalmed10100282

**Published:** 2025-10-01

**Authors:** Nana Akua Abruquah, Obed Kwabena Offe Amponsah, Divya Nair, Douglas Aninng Opoku, Emmanuel Konadu, Kannamkottapilly Chandrasekharan Prajitha, Annabella Bensusan Osafo, Kwame Ohene Buabeng, Nana Kwame Ayisi-Boateng

**Affiliations:** 1 University Hospital, Kwame Nkrumah University of Science and Technology, Kumasi 00233, Ghana; douglasopokuaninng@gmail.com (D.A.O.); emmnl.konadu@outlook.com (E.K.); annabella_bensusan@yahoo.com (A.B.O.); ayisi31@gmail.com (N.K.A.-B.); 2 Department of Pharmacy Practice, Kwame Nkrumah University of Science and Technology, Kumasi 00233, Ghana; okoamponsah@knust.edu.gh (O.K.O.A.); kobuabeng@uhas.edu.gh (K.O.B.); 3 Independent Researcher, Scottsdale, AZ 85259, USA; divsnair08@gmail.com; 4 International Union Against TB and Lung Disease (The Union), 75001 Paris, France; prajitha.kc.consultant@theunion.org; 5 School of Pharmacy, University of Health and Allied Sciences, Ho PMB 31, Ghana; 6 School of Medicine and Dentistry, Kwame Nkrumah University of Science and Technology, Kumasi 00233, Ghana

**Keywords:** antimicrobial stewardship, outpatient, impact assessment, rational use of medicines, operational research, SORT IT, Ghana, INRUD

## Abstract

Rational use of medicines, particularly antimicrobials, is critical for reducing antimicrobial resistance. In 2021, a study conducted at the outpatient department (OPD) of a district hospital in Ghana, identified high antibiotic prescribing and suboptimal adherence to World Health Organization (WHO) prescribing indicators. Based on those findings, antimicrobial stewardship (AMS) intervention was extended to the OPD. This before-and-after study was used to compare WHO prescribing indicators and patterns of antibiotic use, using WHO *AWaRe* (Access, Watch and Reserve) categorization of the years 2021 and 2023. A total of 65,157 patients visited the OPD in 2023 with 171,517 patient encounters and 247,313 prescriptions. Encounters resulting in antibiotic prescriptions halved from 36% to 18%. The average number of medicines prescribed per encounter reduced from three to two. Prescriptions using generic names increased from 76% to 80% and injection use reduced from 7% to 6%. However, prescriptions from the Ghana essential medicines list reduced from 90% to 79%. Access antibiotics use remained unchanged while Watch and Reserve categories increased by 5% and 2%, respectively. The AMS interventions potentially improved three of five WHO indicators. Continued efforts are needed to achieve complete compliance with all indicators and increase access antibiotic use to above 70%.

## 1. Introduction

The World Health Organization (WHO) defines the Rational Use of Medicines (RUM) as, patients receiving medications appropriate to their clinical needs, in doses that meet their own individual requirements, for an adequate period of time and at the lowest cost to them and their community [1].

To measure and monitor drug use in an objective manner, the WHO, in collaboration with the International Network for Rational Use of Drugs (INRUD), developed a set of drug use indicators for hospitals [2]. These indicators serve as a tool to monitor and guide healthcare services for proper documentation of medicine usage with emphasis on prescribing practices, patient care and facility specific factors [2]. These include twelve standardized core drug use indicators, of which five are known as “prescribing indicators” designed to measure whether healthcare providers prescribe drugs appropriately [2]. Each indicator has been assigned an optimum score to allow for objective assessment [3].

In addition to monitoring drug use in general, the indicators also specifically monitor the use of antibiotics, as their irrational use can lead to antimicrobial resistance (AMR). It is recommended that antibiotic prescriptions should be less than 27% of all outpatient prescriptions [4,5]. The prescribing indicator on the use of antibiotics is further enriched by considering antibiotic usage per the WHO *AWaRe* (Access, Watch and Reserve) categories [6,7]. The Access category includes antibiotics for empirical treatment of common infections which should be available in all healthcare settings. The Watch category antibiotics have a higher potential for resistance and their use should be limited. The Reserve category are “last resort” antibiotics and their use should be reserved for special situations with multidrug-resistant bacterial infections where alternative treatments have failed [6,7].

A report from the WHO showed that half of the medicines across the world are prescribed inappropriately, and that half of patients are unable to use their medicines correctly [1]. The World Bank estimates that medicines account for 20–50% of all healthcare expenses in low-and-middle-income countries (LMICs) [8]. Therefore, the RUM is especially important in LMICs such as Ghana that are constrained in terms of resources and effective regulation of the use of medications, while at the same time grappling with AMR [9]. According to the WHO Ghana 2023 annual report, AMR was linked to 25,300 deaths in the country [10]. A situational analysis of antibiotic use and regulation in Ghana conducted in 2017 highlighted that the absence of a national antimicrobial policy, weak regulatory environment and non-compliance to practice standards may have contributed to increased and unregulated access to antimicrobials in Ghana [11].

The Ghana National Drug policy stipulates that there should be routine monitoring of the RUM in healthcare facilities using the WHO core indicators [12]. An operational research (OR) study conducted by Amponsah et al. [13]. in the outpatient department (OPD) of the University Hospital, Kwame Nkrumah University of Science and Technology (KNUST), in 2021 found that only one of the WHO prescribing indicators assessed met WHO optimum levels. In particular, the assessment found antibiotic prescribing to be high at 36%, compared to the WHO recommended threshold of <27% [14,15].

The findings from the OR study were a clarion call to the KNUST hospital management to improve the prescription practices of medicines, especially antibiotics in the OPD. Following the assessment, an antimicrobial stewardship (AMS) program which had been instituted for inpatients was extended to include the outpatient department.

Assessing the impact that these interventions may have made on prescriber compliance to WHO standards of prescribing is imperative, so that informed decisions can be taken to improve the rational use of medicines including antibiotics at the OPD of the hospital. Considering the assessment of 2021 as a baseline, this study aimed to assess if there has been any change in drug use as per the WHO prescribing indicators in the OPD of the University Hospital, KNUST. The specific objectives were to (among the prescriptions of all the patients who sought care at the OPD of the University hospital during the calendar year of 2021 (first OR study) and 2023 (current OR study)).

i. compare the key WHO prescribing indicators;
ii. compare the pattern of antibiotic prescriptions according to the WHO *AWaRe* classification.

## 2. Materials and Methods

### 2.1. Study Design

This study was a comparison of compliance to WHO rational prescribing guidelines using routinely collected data from the Electronic Medical Records (EMRs) of outpatients at the University Hospital, KNUST, measured through cross-sectional assessments conducted during 2023 (current study) and 2021 (first OR study).

### 2.2. General Settings

Ghana is a West African country bordered by Burkina Faso to the north, the Gulf of Guinea to the south, Togo in the east and Cote D’Ivoire in the west [16]. Administratively, the country is divided into sixteen regions with their own regional capitals. Healthcare services in the country are delivered through private and public facilities [11]. The public facilities include Health Centers, District Hospitals, Regional and Tertiary hospitals [17]. District hospitals form an important aspect of healthcare provision as the first point of referral [11]. Medicines prescribed through the National Health Insurance Scheme are dispensed free of charge at health facilities [18]. Clients can buy medicines including antibiotics, from pharmacies and over the counter (OTC) medicine sellers (for OTC medicines) through out-of-pocket payments.

### 2.3. Specific Settings

The University Hospital, KNUST, is located in the second largest city in Ghana, Kumasi, the capital of the Ashanti region [16]. It is a 135-bed capacity quasi-government district-level hospital serving primarily the Oforikrom Municipality with approximately 303,016 inhabitants [16]. The facility also tends to patients from the municipality’s environs, extending to Northern Ghana. The University Hospital offers primary care services to residents in the municipality and receives referrals from other health facilities. The average OPD footfall per day was 136 patients in 2021.

The point of entry for individuals seeking healthcare at the University Hospital is the OPD. The individuals are checked into the hospital by a receptionist from the Records Department. An account is activated for the individual through the EMRs—a web-based electronic medical recording system which connects various departments of the hospital. The hospital attendant is subsequently triaged by a registered nurse and directed to see a prescriber in a consulting room.

A new electronic hospital data management system has been operationalized since December 2022. The current EMR system has modules including an outpatient module with different levels of access granted based on the cadre of the healthcare provider. The demographic data and clinical records including antibiotic prescriptions of patients attending the facility can be accessed using a unique patient identification number.

### 2.4. Process of Prescription and Issuing of Medicines to Outpatients in the University Hospital, KNUST

The medical prescription following the patient encounter is directly documented into the EMR system by the consulting prescriber (medical doctor, dentist, physician assistant, ophthalmic nurse, dialysis nurse and dental nurse). Treatment advice is immediately received at the pharmacy department via the EMR for dispensing the medicines to the patient. For medicines unavailable at the facility, a paper-based electronic prescription is generated and provided to the patient for purchase from other pharmacies.

### 2.5. Antimicrobial Stewardship (AMS) Activities Undertaken at the University Hospital, KNUST, After the First Operational Research Study

The results of the baseline operational study were disseminated using multiple communication tools targeting decision makers and stakeholders of the University Hospital. This led to the extension of the AMS program to the OPD from November 2022, which was previously operationalized in the inpatient department only. Under the AMS program, the healthcare providers were trained in capacity building on rational antibiotic use and infection prevention and control. Measures were undertaken to ensure the documentation of indications for antibiotic prescription through routine monitoring by the AMS team. Healthcare providers were informed and trained to conduct culture and drug susceptibility analyses before the initiation of empiric antibiotic therapy. In consultation with specialists in the hospital, certain antibiotics were placed on restricted access (requiring prior authorization before use) to reduce their inappropriate use. Dissemination details (Table A1) and implementation status of the recommendations (Table A2) of the first OR study are provided in Appendix A.

### 2.6. Study Period

The first OR study was conducted utilizing the data collected between January and December 2021. The current study was conducted using data collected between January and December 2023. Data analysis and compilation of results were performed between January and March 2025.

### 2.7. Study Population

The study population in the first OR study and the current study included the prescriptions of all patients who sought care at the OPD of the University Hospital during the calendar years 2021 and 2023.

### 2.8. Sample Size

We considered all the prescriptions from both years (2021 and 2023) of patients attending the OPD of the hospital.

### 2.9. Data Collection and Data Variables

The data for this study were extracted from the EMR database by the University IT team. The demographic and clinical characteristics of patients who sought care during the calendar year 2023 were extracted. To compare the compliance with WHO prescribing indicators, the name and class of antibiotics, number of antibiotics prescribed in each patient encounter and route of administration of the medicines were extracted from two calendar years, 2021 and 2023.

### 2.10. Data Analysis

Data were retrieved in MS Excel format and analyzed using STATA^®^ (version 16.0 Copyright 1985–2019, StataCorp LLC, College Station, TX, USA). The demographic and clinical characteristics of patients were summarized using frequencies and percentages.

Calculation of indicators was performed, as shown in Table 1. Each indicator was summarized as a percentage. The indicator “Average number of medicines per patient encounter” was summarized as mean. Each indicator was compared between the first OR study and the current OR study using a test of two proportions along with a 95% confidence interval (95% CI) for the change in proportions. A *p*-value < 0.05 was considered significant.

### 2.11. Operational Definitions

A consultation with a single patient on a single day in a department of the OPD was considered as a patient encounter. Each medicine prescribed during a patient encounter was considered as a separate prescription. A patient was considered to have been prescribed an antibiotic if any of the prescriptions retrieved against their unique identifier contained an antibiotic. Antibiotics prescribed were categorized according to the WHO *AWaRe* classification 2023 into the Access, Watch, or Reserve group [19]. The medicines in the prescription were matched with the medicines listed in the Ghana EML to identify whether they were listed in the EML or not.

## 3. Results

Between January and December 2023, 65,157 patients visited the OPD of the KNUST Hospital and recorded 171,517 patient encounters. In comparison, during the same period in 2021, 49,660 patients accounted for 110,280 patient encounters. In 2023, 151,823 total patient encounters led to prescription of at least one medicine. In total, there were 247,313 prescriptions in 2023 compared to 350,149 prescriptions in 2021.

### 3.1. Demographic and Clinical Characteristics

More than half of the patients in 2023 were female (56.1%) and approximately 45% were between the age group of 15 and 24 years. The median (interquartile range) age of patients was 22 (19–37) years. Table 2 shows the demographic and clinical characteristics of patients who attended the OPD of the University Hospital, KNUST in 2021 and 2023. The general OPD services accounted for about 79% and 86% in 2021 and 2023, respectively. There was a statistically significant (*p* < 0.001) increase in the proportion of patients with a single encounter in 2023 compared to 2021 (12.3% to 41.9%). Also, the proportion of patients with multiple encounters (three and more) decreased in 2023, compared to 2021 (73% to 38.4%).

### 3.2. Comparison of Adherence to WHO Prescribing Indicators Between 2021 and 2023

**Figure 1:**
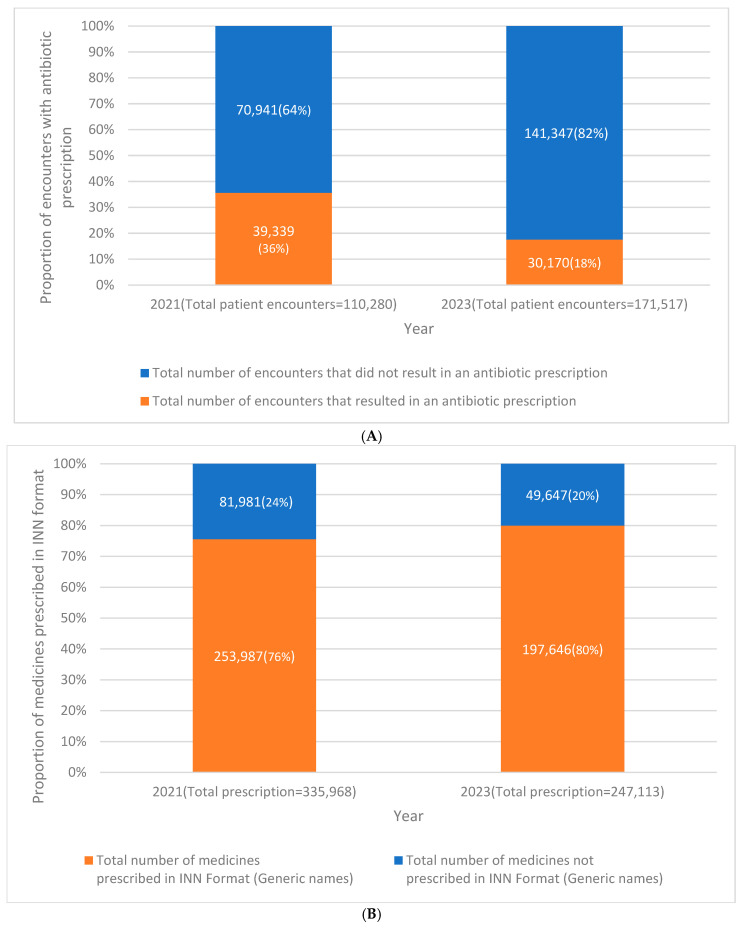

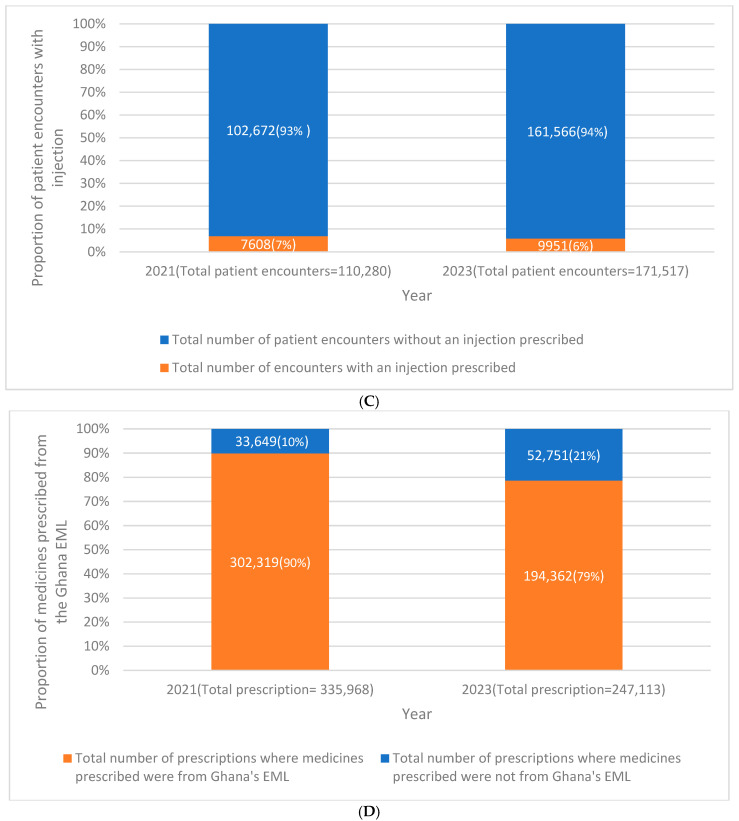
(**A**) Comparison of the number and proportion of patient encounters that resulted in an antibiotic prescription in the outpatient department of the University Hospital, KNUST, in Ghana between January and December 2021 compared with January– to December 2023. Percent change (2023 vs. 2021) = 18.1% (95% confidence interval: 17.7–18.4%, *p*-value: <0.001). (**B**) Comparison of the number and proportion of medicines prescribed in INN (International Non-proprietary Names) format (generic names) in the outpatient department of the University Hospital, KNUST, in Ghana between January and December 2021 compared with January to December 2023. Percent change (2023 vs. 2021): −4.4% (95% confidence interval: −4.2 to −4.6%, *p*-value: <0.001). (**C**) Change in number and proportion of patient encounters with an injection prescription in the outpatient department of the University Hospital, KNUST, in Ghana between January and December 2021 compared with January to December 2023. Percent change (2023 vs. 2021) = 1.1% (95% confidence interval: 0.9–1.3%, *p*-value: <0.001). (**D**) Change in number and proportion of medicines prescribed from Ghana’s essential medicines list (EML), 2017 in the outpatient department of the University Hospital, KNUST, in Ghana between January and December 2021 compared with January to December 2023. Percent change (2023 vs. 2021) = 11.3% (95% confidence interval: 11.1–11.5%, *p*-value: <0.001). Figure 1A–D Change in WHO prescribing indicators in the outpatient department of the University Hospital, KNUST, in Ghana between January and December 2021 compared with January to December 2023.

The proportion of patient encounters that resulted in an antibiotic prescription decreased significantly from 36% in 2021 to 18% in 2023 (95% CI: 17.7–18.4%, *p*-value < 0.001) (Figure 1A). The average number of medicines prescribed per encounter reduced to two (*p* < 0.001), ranging from 1 to 21 compared to three in 2021, with a range of 1–18. The proportion of medicines prescribed using generic names increased from 76% in 2021 to 80% in 2023 (95% CI for increase: 4.1–4.5%), and this was statistically significant. (Figure 1B). Compared to 2021, in 2023, the prescription of injections reduced by 1.1% (95% CI: 0.9–1.3%, *p* < 0.001) (Figure 1C). Figure 1D shows that 79% of the medicines prescribed in 2023 were from the Ghana essential medicines list compared to 90% in 2021.

Values in each figure may add up to more than 100% due to rounding to the nearest whole number.

### 3.3. Pattern of Antibiotic Prescriptions According to the WHO AWaRe (Access, Watch, Reserve) Classification in 2021 and 2023

Table 3 shows the pattern of antibiotic prescriptions by the WHO Access, Watch and Reserve categories in 2021 and 2023. In both years, approximately 48% of the antibiotics were prescribed from the Access group. The prescription of antibiotics from the Watch group and Reserve group increased by 4.6% (46.5% in 2021 to 51.1% in 2023) and 1.7% (1.8% in 2021 to 3.5 in 2023), respectively. The prescription of antibiotics that belong to the Not recommended category reduced from 3.1% in 2021 to 2.6% in 2023. These differences were found to be statistically significant (*p* < 0.001). Table A3 shows the pattern of antibiotic prescribing according to the WHO *AWaRe* classification in 2023.

## 4. Discussion

The present comparative study demonstrated an improvement in the compliance with WHO indicators compared to the baseline OR study conducted in 2021 in the district-level hospital in Ghana [14,15]. Three prescribing indicators changed in the hospital OPD setting following the extension of the AMS intervention as a result of the baseline study. First, there was a reduction in the proportion of patient encounters that resulted in antibiotic prescriptions to 18% in 2023 from 36% in 2021. Second, the average number of antibiotics prescribed per encounter was reduced from three to two. Finally, the prescription of injections was reduced further to 6% from 7%. Hence, among the five WHO prescribing indicators, two have achieved levels within the optimum recommended by the WHO.

Our study has several strengths. First, the current study is built on baseline OR, and the use of cross-sectional assessment before and after the intervention has enabled a clear assessment of changes in key indicators. Second, the use of EMR data with very few missing data minimized reporting bias and allowed robust evaluation. Finally, we have adhered to the Strengthening the Reporting of Observational Studies in Epidemiology (STROBE) guidelines for reporting this study [20]. The study has a few limitations. We did not have a control group or setting to compare to, so it is difficult to completely rule out the effect of other external factors that might have influenced the observed changes. Hence, the changes observed cannot be completely attributed to the extension of the AMS intervention. Another limitation of our study is that we conducted this study in a single district-level hospital; hence, the findings may not be generalizable to all other settings, particularly rural or private facilities in Ghana. This is because, the current study site is located in an urban area in addition to being a quasi-governmental facility which may not reflect the setting of most health facilities in Ghana. Expanding this study to a representative sample of facilities in the country would enable the generation of generalizable findings relevant to the country. Further, the data structure downloaded from the EMRs did not lend itself to statistical techniques like regression, due to which the impact of demographic and clinical characteristics on the prescribing indicators assessed in the different years could not be assessed. The authors also acknowledge that the large sample size used for both years may exaggerate the impression of improvements seen. Subsequent assessments could include additional assessments to further explore the factors and determinants of the findings.

The study has several policy and practical implications too, for AMR stewardship in the hospital setting. The OPD is often the first point of contact for most patients in a hospital setting in LMICs. However, the AMS program (ASP) in hospitals is often implemented in inpatient settings. The evidence on their impact in OPDs, particularly in low-and-middle-income countries (LMICs), remains limited compared to high-income countries [21,22] and our study may help reduce this evidence gap. With the study findings, we highlight the feasibility and utility of extending ASP activities beyond inpatient settings, which is the first and foremost implication of our study.

Secondly, the improvements in the WHO prescribing indicators observed in 2023 may be due to capacity-building interventions, routine audits and feedback under the ASP, implemented and extended to the outpatient setting of the hospital [13]. These improvements align with broader efforts seen across African countries, where hospitals are increasingly adopting ASP to improve antibiotic use [13,23,24]. Evidence from antibiotic use assessments across hospitals in Ghana, Uganda, Zambia and Tanzania also highlight the high empirical use of antibiotics, emphasizing the critical need for strengthening AMS interventions in hospital settings [25,26,27]. Our study thus aligns with the growing momentum for AMS across the continent.

Third, our study has utilized the WHO’s key prescribing indicators. This has offered insights into their relevance to local practice and to understanding whether the international benchmarks are valid in our setting. However, we recommend further research in this area to evaluate its appropriateness and contextual fit of these indicators. In addition, implementation of AMS using practical toolkits, such as those developed by the WHO and the Commonwealth Partnerships for Antimicrobial Stewardship (CwPAMS) [28,29], in achieving measurable improvements, helps us to understand how these can be adopted in our setting.

Further, analysis of antibiotic prescription patterns using the WHO *AWaRe* classification demonstrated that the proportion of antibiotics from the Access category remained unchanged in 2021 and 2023. The WHO recommends a target of 70% and above for the Access category of antibiotics which was not met in the hospital [30]. Prescribers had requested the list of antibiotics in line with the *AWaRe* classification in the consulting rooms to improve ease of use, which had not been completed. This may account for the non-compliance observed and leaves room for continuous quality improvement in prescribing through the ASP. The WHO *AWaRe* antibiotic book, which was introduced to improve antibiotic use, has not been widely disseminated among prescribers in the hospital which potentially could have improved compliance [31]. Similar patterns have been documented in several African countries, with empirical prescribing and limited microbiological support contributing to suboptimal *AWaRe* distribution, especially with high use of Watch antibiotics [32]. In some LMIC outpatient hospital settings, Watch group antibiotics have been reported to constitute as high as 60% of total prescriptions [33]. Even though the increase in Watch and Reserve group antibiotic use in our study may potentially be due to clinical necessity in some cases, it is important that continued monitoring and training be conducted due to the higher potential of these categories to drive antimicrobial resistance [6,19].

These improvements aside, compliance to the other prescribing indicators reduced in 2023 compared to 2021. Prescribing from the EML reduced by 11% which may be due to a few reasons. The education provided to health workers as part of the intervention did not include any material or reminders on prescribing from the EML which may have accounted for this finding. Another potential reason may be the fact that the Ghana EML has not been updated since 2017, which may not reflect the latest global evidence from the WHO and may impact prescriber perceptions and use [34]. Prescribing medicines with their generic names also increased by 4% in the second assessment, which is still 20% less than the optimum. The hospital primarily serves members of the University community who receive medicines for free and can request specific brands to be prescribed on the EMR for them. This may account for this indicator not reaching the optimal levels and needs work in terms of training and education. Additionally, the developers of the EMR may be engaged to only allow prescriptions in the generic name but allow the dispensing to be switched to a brand on request at the pharmacy. Future training programs should be designed to include principles of prescribing in compliance with the WHO indicators.

This study highlights the potential improvements in indicators beyond the prevalence of antimicrobial use and antimicrobial consumption rates in low resources settings like Ghana. For the impact on WHO prescribing indicators, AMS interventions such as education and training may need to include materials that cover these indicators to ensure compliance improves with AMS implementation. With the large proportion of OPD attendance and potential for widespread antimicrobial use, AMS interventions are needed in outpatient settings to impact prescribing and mitigate the spread of AMR.

## 5. Conclusions

The study has demonstrated the importance of operational research, its findings and impacts in real-world patient care settings. The AMR stewardship intervention instituted has significantly improved the rational use of antibiotics and increased compliance with WHO quality standards for prescribing at the OPD of the University Hospital. However, targeted efforts are still needed to further enhance prescribing of the Access group and minimize the reliance on Watch and Reserve antibiotics unless clinically justified, as part of sustained interventions to contain antimicrobial resistance. Also, a qualitative exploration to identify enablers and barriers to the rational use of antibiotics at the hospital would further guide the scale up and sustainability of the interventions.

## Figures and Tables

**Table 1:** Calculation of World Health Organization (WHO) prescribing indicators for drug use.

Indicator	Numerator	Denominator	WHO Optimum
Proportion of patient encounters that result in an antibiotic prescription	Number of encounters in which an antibiotic was prescribed	Total patient encounters for which data were collected	<27%
Average number of medicines per patient encounter	Total number of medicines prescribed	Total patient encounters for which data were collected	<2
Percentage of medicines prescribed by generic name	Total number of medicines prescribed in International Non-proprietary Names (INN) format	Total number of medicines prescribed in all patient encounters for which data were collected	100%
Percentage of encounters with an injection prescribed	Number of encounters in which an injectable form of medicine was prescribed	Total patient encounters for which data were collected	<20%
Percentage of medicines prescribed from an essential medicines list	Total number of medicines prescribed from the EML	Total number of medicines prescribed	100%

**Table 2:** Demographic and clinical characteristics of patients attending the OPD of the University Hospital, KNUST, January–December 2023 compared with January–December 2021.

Characteristics	2021		2023		*p*-Value
	Number	(%)	Number	(%)	
**Total number of patients**	49,660		65,157		
**Demographic characteristics**					
**Age in years ^1^**					
0–14	6301	(12.7)	8638	(13.3)	<0.001
15–24	22,705	(45.7)	29,127	(44.7)	
25–34	7501	(15.1)	9433	(14.5)	
35–44	4004	(8.1)	5784	(8.9)	
45–54	3312	(6.7)	4365	(6.7)	
55–64	2867	(5.8)	3526	(5.4)	
≥65	2964	(5.9)	4280	(6.6)	
**Sex**					
Male	26,783	(53.9)	28,592	(43.9)	<0.001
Female	22,877	(46.1)	36,565	(56.1)	
**Clinical characteristics**					
General OPD	39,186	(78.9)	56,026	(86)	<0.001
Dental	2108	(42.0)	2286	(3.5)	
Diabetes/Hypertension clinic	517	(1.0)	360	(0.6)	
Others ^2^	7849	(15.8)	6485	(10)	
**Patient encounters**					
1	6108	(12.3)	27,306	(41.9)	<0.001
2	7263	(14.6)	12,828	(19.7)	
3	10,542	(21.2)	10,406	(15.9)	
4	9827	(19.8)	7073	(10.9)	
5	7516	(15.1)	4051	(6.2)	
6	4287	(8.6)	1890	(2.9)	
>6	4117	(8.3)	1603	(2.5)	

^1^ Implausible value in age for six patients in 2021 and four patients in 2023 were excluded, ^2^ others include specialty services like antenatal services, dialysis services, ENT services, eye services, general surgery services, orthopedic and urology services, OPD—outpatient department, KNUST—Kwame Nkrumah University of Science and Technology.

**Table 3:** Pattern of antibiotic prescriptions according to the WHO *AWaRe* (Access, Watch, Reserve) classification in the outpatient department of the University Hospital, KNUST, in Ghana in January-December 2021 compared with January-December 2023.

Group	2021		2023		% Change ^2^	(95% CI)	*p*-Value
	Number of Antibiotic Prescriptions (*n* = 58,885)	(%) ^1^	Number of Antibiotic Prescriptions (*n* = 37,646)	(%) ^1^			
Access group	28,152	(47.8)	18,013	(47.8)	0	-	0.9
Watch group	27,395	(46.5)	19,237	(51.1)	4.6	(3.9–5.2)	<0.001
Reserve group	1045	(1.8)	1308	(3.5)	1.7	(1.4–1.9)	<0.001
Not recommended (Ciprofloxacin-Tinidazole combination)	1798	(3.1)	969	(2.6)	−0.5	(−0.7–−0.3)	<0.001
Unclassified ^3^	495	(0.8)	601	(1.6)	0.8	(0.6–0.9)	<0.001

^1^ Percentages were calculated with total number of antibiotic prescriptions as the denominator. Whenever a prescription included a fixed-dose combination of antibiotics, each of the component antibiotics were classified into their respective *AWaRe* categories and therefore % may not add to 100. ^2^ % change calculated as 2023–2021. ^3^ Unclassified includes Bacitracin and Mupirocin which are not listed in the *AWaRe* list and those prescriptions which mentioned “antibiotic” without specifying the name of the antibiotic. WHO—World Health Organization, KNUST—Kwame Nkrumah University of Science and Technology, CI—confidence interval.

## Data Availability

Requests to access these data should be sent to the corresponding author.

## Appendix A

### Appendix A.1. Dissemination Details of the Baseline Operational Research Study

**Table A1:** Dissemination details of the baseline operational research study at the University Hospital, KNUST in 2021.

How	To Whom	Where (Number)	When
Stakeholder engagement and communication planning	Officials of KNUST Director, University Health Services Clinical training coordinator Pharmacy Superintendent Antimicrobial Stewardship Committee, University Hospital, KNUST	University Hospital, KNUST (13)	October 2021, June–August; October–December 2022,
Evidence summaries (handouts summarizing study findings)	Healthcare providers and students of KNUST	Newsstand of the Dean’s office of the School of Pharmacy, KNUST (Approximately 15 staff and more than 100 students)	October 2022 to July 2023
	Seminar attendees and via email	Ghana SORT IT AMR dissemination Seminar (36)	July 2023
PowerPoint presentation and discussions	The study findings were disseminated during various training seminars conducted for staff of the University Hospital, KNUST and the students at the faculty of Clinical Pharmacy	Department of Pharmacy Practice, Faculty of Pharmacy and Pharmaceutical Sciences, KNUST (10)	December 2022–January 2023
		School of Graduate Studies, KNUST (approximately 35)	July 2023 to present (May 2024)
Manuscript full text dissemination via emails	Representatives of ReAct Africa (Action on Antibiotic Resistance) AMR Technical Office, Ministry of Health, Ghana Ghana AMR Coordinating Committee		August 2022
Publication in a peer-reviewed journal	International Journal of Environmental Research and Public Health (IJERPH)	Reference 1 [14] (2157 article views according to IJERPH; 16 citations according to Google scholar) Reference 2 [15] (1582 article views according to IJERPH; 2 citations according to Google scholar)	August 2022 to present September 2022 to present

KNUST—Kwame Nkrumah University of Science and Technology, SORT IT—Structured Operational Research Training Initiative, AMR—antimicrobial resistance.

### Appendix A.2. Recommendations and Actions Taken Following the Baseline Operational Research Study

**Table A2:** Recommendations and actions taken following the baseline operational research study at the University Hospital, KNUST in 2021.

Recommendation	Action Status	Details of Action (When and What)
EMR is customized to control the prescription of Watch and Reserve antibiotics only to higher ranked prescribers and medical conditions in consultation with pharmacists	Partially implemented and ongoing	*December 2022–present* EMR changed to different type of management software. Director of Hospital in talks with software managers to incorporate custom controls on antibiotic prescriptions into new software
OPD services included in the antimicrobial stewardship program	Implemented	*July 2023–present* Current iteration of antimicrobial stewardship activities including point prevalence surveys include outpatient services in purview, staff education and capacity building seminars.
Six-monthly audit and feedback system for OPD prescriptions have been established	Implemented	*Nov 2023, May 2024* On-going online point prevalence surveys to audit prescriptions
Drafting of an antibiotic prescription policy document on antibiotic prescribing and use at the hospital	Implemented	*September 2023* A policy document has been developed which covers outpatient and inpatient antibiotic use and the role of all the health cadres in the hospital in prescribing. Provisional approval has been given, awaiting formal adoption by Hospital Management Committee
Additional research is planned with hospital management as a follow-up on outpatient and inpatient antibiotic use practices in the hospital.	Partially implemented and ongoing	*November 2023–present* Inpatient and outpatient point prevalence surveys ongoing. Routine audits of prescriptions on going. Publication for antibiogram developed on account of activities undertaken by AMS team under review by JAC-Antimicrobial Resistance
Training seminars on AMR, AMS, and IPC organized for all health cadres at the hospital	Implemented and ongoing	*July 2023–present* Since inception of the AMS committee, various training seminars have been organized for staff of the university hospital on AMR. Newly appointed staff orientation exercise also includes AMR/AMS as important topic so that all staff are kept abreast of guidance before starting official duties at the hospital
Additional funding for AMS, laboratory infrastructure and research	Partially implemented and ongoing	*June 2023* Expansion of microbiology laboratory and acquisition of new equipment at the university hospital to conduct culture and sensitivity testing

EMR—electronic medical records, OPD—outpatient department AMS—antimicrobial stewardship, IPC—infection prevention and control, AMR—antimicrobial resistance.

**Table A3:** Distribution of antibiotic prescriptions according to WHO *AWaRe* classification at the University Hospital, KNUST, 2023.

WHO *AWaRe* Group	Antibiotic	*n*	Percentage
Access	Ampicillin	1	0.003
	Chloramphenicol	13	0.033
	Amikacin	87	0.222
	Amoxicillin Clavulanic Acid	9327	23.823
	Clindamycin	1283	3.277
	Co-Trimoxazole	28	0.072
	Doxycycline	801	2.046
	Flucloxacillin	869	2.22
	Gentamicin	161	0.411
	Metronidazole	3165	8.084
	Nitrofurantoin	21	0.054
	Secnidazole	443	1.131
	Tetracycline	575	1.469
	Benzylpenicillin	270	0.69
Watch	Azithromycin	2953	7.542
	Cefaclor	1	0.003
	Cefixime	393	1.004
	Cefotaxime	1	0.003
	Cefpodoxime	62	0.158
	Ceftriaxone	1007	2.572
	Cefuroxime	3964	10.125
	Ciprofloxacin	6869	17.544
	Clarithromycin	365	0.932
	Erythromycin	105	0.268
	Fusidic Acid	1	0.003
	Levofloxacin	880	2.248
	Neomycin	1310	3.346
	Meropenem	8	0.02
	Ofloxacin	7	0.018
	Tobramycin	1302	3.326
	Vancomycin	1	0.003
	Fosfomycin	1	0.003
Reserve	Polymixin	1308	3.341
Not Recommended	Ciprofloxacin/Tinidazole	969	2.475
Unclassified	Mupirocin	1	0.003
	Bacitracin	600	1.532
